# Melt Pool Shape Evaluation by Single-Track Experiments and Finite-Element Thermal Analysis: Balling and Lack of Fusion Criteria for Generating Process Window of Inconel738LC

**DOI:** 10.3390/ma16041729

**Published:** 2023-02-20

**Authors:** Jun Katagiri, Masahiro Kusano, Satoshi Minamoto, Houichi Kitano, Koyo Daimaru, Masakazu Tsujii, Makoto Watanabe

**Affiliations:** 1 Integrated Smart Materials Group, Research Center for Structural Materials, National Institute for Materials Science, Tsukuba 305-0047, Japan; 2 Materials Integration System Team, Research and Service Division of Materials Data and Integrated System, National Institute for Materials Science, Tsukuba 305-0044, Japan; 3 Welding and Joining Technology Group, Research Center for Structural Materials, National Institute for Materials Science, Tsukuba 305-0047, Japan

**Keywords:** additive manufacturing, laser powder bed fusion, process window, finite-element method, thermal conduction, single-track experiment, balling, lack of fusion, Inconel738LC, materials integration

## Abstract

Defects occur in laser powder bed fusion (L-PBF) such as the keyholing, lack of fusion, and the balling depending on the laser power (*P*) and the scan speed (*V*). The figure shows that the occupied regions of each defect are the process window and are essentially important to fabricate a high-quality part. This paper is a study of process window generation using single-track experiments and finite-element method simulation of thermal conduction for Inconel738LC alloy. A series of single-track experiments were conducted varying the range of *P* and *V* and the results were classified into keyholing, lack of fusion, balling, and good track. A series of simulations were conducted and validated by comparison with the experiments. To quantitively identify the balling, the isolines from the contour map generated by the results of simulations and the balling criteria of the ratio of melt pool length and the depth (*L*/*D*) of 7.69 were determined considering the past theoretical studies. The lack of fusion criteria: the ratio of the overlap depth in fabrication using multi-scan (*D*_ov_) and powder layer thickness (*t*) of 0.1 was obtained. Using the criteria obtained from the experiments and simulation, the process window was generated.

## 1. Introduction

Inconel738LC is a nickel-based alloy. Advantages of Inconel738LC are high heat resistivity and high corrosion resistance, while a disadvantage is its hard workability using conventional machining devices. An optimum design technology can provide a novel design to achieve higher performance than the conventional design, e.g., [1]. Such novel and unique design is often not manufacturable using the conventional machining devices. Additive manufacturing, especially, the laser powder bed fusion (L-PBF) process enables us to manufacture the novel design obtained from the optimum design. Hence, the L-PBF process is one of the promising additive manufacturing processes and its technical development is rapidly increasing all over the world. During the SLM process, the powder layer is thinly spread on the substrate. The unit shaping process in the L-PBF is that the moving laser melts the powder layer and then solidifies. An arbitrary-shaped part can be fabricated by repeating the unit shaping process. An advantage of the L-PBF is to fabricate a complex-shaped part. 

The laser power and the scan speed strongly influence the melt pool geometry during the L-PBF process. According to the past studies, Seede et al. quantitatively evaluated the melt pool geometry of AF9628 alloy by a series of single-track experiments varying the laser power and the scan speed [2]. When the laser power and the scan speed are relatively high, the melt pool forms an elongate shape; in such case, the melt pool is split into spherical droplets due to Plateau–Rayleigh capillary instability [3,4]. Such defect is known as balling [5,6,7,8,9,10]. With low laser power and/or high scan speed, the powder layer melts insufficiently; hence, the pores in the powder particle assembly remain after passing the laser. Such type of defect is known as lack of fusion [2,11]. When there is high laser power and the low scan speed, the melt pool reaches a deep point of the substrate. In such a case, the front end of the laser-excavated region breaks into pore droplet(s). Such defect is called a keyhole pore or simply keyholing [12,13,14]. It is quite important to identify the regions of the keyholing, lack of fusion, balling, and the good track region in the laser power and the scan speed (*P*-*V*) space. Note that the good track is a region in the *P*-*V* space when such defects do not occur. The figure which depicts the occupied regions of such defects in the *P*-*V* space is known as the process window or process map. The process window is essentially important to fabricate a part by the L-PBF process. 

**Figure 1:**
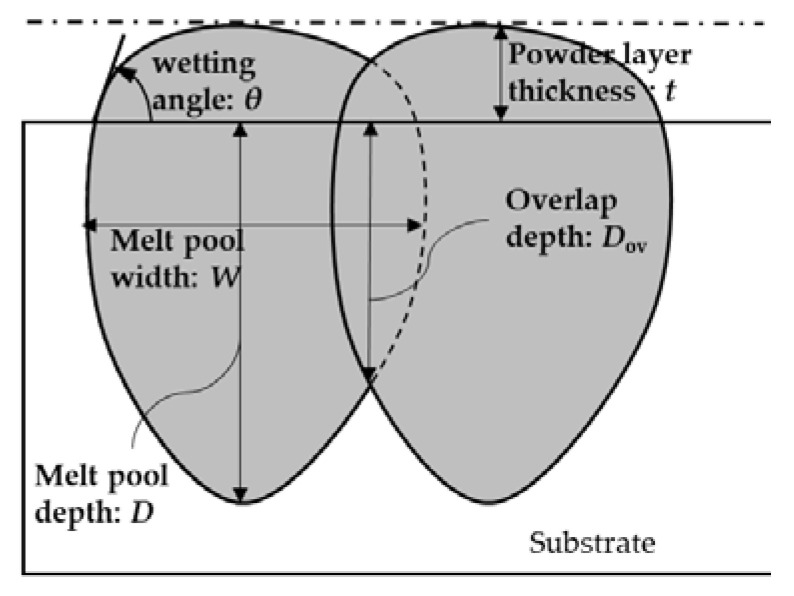
A schematic of the extended Eager–Tsai (E-T) model for the multi-scans in the L-PBF process.

A series of single-track experiments and the image analysis of the cross-sectional images are required to obtain the process window. The melt pool depth (*D*) and width (*W*) are evaluated by the cross-sectional image analysis. The melt pool shape is characterized using the *D* and *W*. Eager and Tsai developed a simple geometrical model for predicting melt pool geometry [15]. We refer to the geometrical Eager–Tsai (E-T) model. Multiple scans are applied in the L-PBF process; however, the original E-T model is for predicting the melt pool geometry of a single scan in the welding process. Seede et al. extended the E-T model to apply to the L-PBF process by incorporating the hatching distance (*h*) between the two scans [2]. A schematic of the extended E-T model is shown in Figure 1. 

The overlap depth (*D*_ov_) is the depth of overlap area between the first and the second laser scans.

According to the past studies, *D*/*W* and *D*/*t* have been used to identify keyholing and lack of fusion, respectively [2,11,16,17]. Note that *t* is the powder layer thickness. The keyholing defect was observed when *D*/*W* > 0.5–0.83 [2]; however, the authors claimed that the criterion was somewhat low because the range of laser powers was relatively low in their experiments; hence, the authors used the criterion *D*/*W* > 2.2 for considering the simple geometrical melt pool model proposed by Eager and Tsai [15]. Roehling et al. conducted experiments using Ti-Nb alloys and obtained the keyholing criterion of *D*/*W* > 1.5 [18]. Kitano et al. conducted single-track experiments using the Ni-based alloy of Hastelloy X and used the keyholing identification by *D*/*W* > 1.0. The lack of fusion has been identified using the criterion *D* < *t* [2,11,17]. The meaning of *D*/*t* < 1 is clear; the powder layer insufficiently melts by the single-track experiment. However, the part is fabricated by multiple scans; the melt pools of the first and the second scans overlap. In such case, the depth of the overlap region is rather important for evaluating the lack of fusion criterion. Seede et al. [2] proposed a geometrical relation of the overlap depth (*D*_ov_) using the hatching distance (*h*), *D*, and *W* based on the Eager–Tsai melt pool model [15]. The *D*_ov_/*t* will be preferable compared to *D*/*t* for the lack of fusion criterion. 

Balling is difficult to identify by the *D* and the *W*. Balling has been identified by the distinctive features obtained from cross-sectional images [2,17] and top-view pictures [2]. According to past studies, Plateau–Rayleigh capillary instability is the phenomenon that the cylindrical fluid stream breaks up small droplets, and causes balling [3,16]. When there is high laser power and high scan speed, the melt pool forms an elongated shape, which implies that the melt pool length (*L*) is essential. This implies that Plateau–Rayleigh instability arises when there is a large *L* and small radius if the melt pool is a cylindrical shape. Gusarov and Smurov determined the necessary and sufficient condition for Plateau–Rayleigh instability to arise assuming that the melt pool was an infinitely cylindrical shape [3]. Following the study of [3], DebRoy et al. proposed the criterion *L*/*D* > π [16]. Except for these studies, authors have hardly found the criterion value of *L*/*D* in the literature. 

To obtain melt pool geometry parameters such as *D*, *W*, and *L*, computational fluid dynamics (CFD) analysis incorporating thermal conduction, phase change between solid, liquid, and gas, and the moving laser heat source model have been extensively conducted in the last decade, e.g., the studies of [19,20,21]. An advantage of the CFD analysis is to quantitatively evaluate the melt pool geometric parameters, while a disadvantage is the huge computational load, i.e., the long computation time. For example, the laser drilling (i.e., the laser does not move) simulation of Ti-6Al-4V alloy requires about 24 h [22]. The powder bed was not incorporated in the study of [22]. The computational load becomes high when the simulation incorporates powder particle assembly. To shorten the computation time, the finite-element method simulation was applied with two assumptions: (1) the powder bed is modeled as continuum media and (2) substituting Beer–Lambert absorption law for the ray-tracing method [23,24]. Note that the ray-tracing method can precisely model the laser–material interaction and has been used in the past for CFD simulation of L-PBF [25]. The computation time using the simulation in the study of [23,24] is approximately 3.5 times less than that of the other simulation models [24]. However, the computational load of the CFD simulation is still high because many simulations are required to generate the process window. 

**Figure 2:**
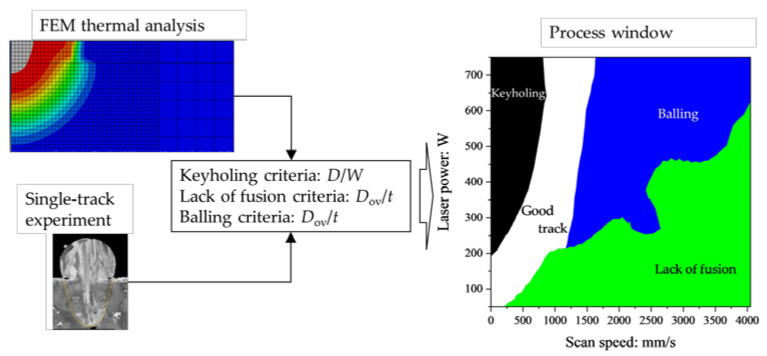
A workflow to generate the process window using the results from FEM simulation and single-track experiment.

The purpose of this study is to determine the balling and the lack of fusion criteria using the single-track experiments and the finite-element method simulation of thermal conduction for Inconel738LC alloy. The single-track experiments and the FEM analysis procedures are detailed in Section 2. In Section 3, the results of the experiments and the FEM simulations are discussed with the criteria for identifying balling and lack of fusion. As a result, the balling criterion of *L*/*D* > 7.69 and the lack of fusion criterion of *D*_ov_/*t* < 0.1 were obtained. Moreover, as shown in Figure 2, the framework to generate the process window using the FEM simulations and the criteria for keyholing, lack of fusion, and balling are developed in Section 3. Finally, the conclusion of this study and the future investigation are described in Section 4. 

## 2. Materials and Methods

### 2.1. Single-Track Experiment

The material used in this study was Inconel738LC. The L-PBF system of SLM280HL (SLM Solutions, Lübeck, Germany) was employed for the single-track experiments. The Inconel738LC powders (Amerprint^®^0151.074, Höganäs AB, Höganäs, Sweden) made by the gas atomized method were used. Note that the substrate under the powder layer was also Inconel738LC. The mean particles diameter of Inconel738LC was about 28.9 μm. The powder was thinly coated at the surface of substrate. The powder layer thickness (*t*) was about 50 μm. A series of the single-track experiments was conducted under the various laser powers and scan speeds (translation velocity of moving laser) listed in Table 1. In this study, just the laser power and the scan speed were varied in the single-track experiment. Note that the melt pool geometry depends on the other factors such as the laser beam profile (Gaussian, flat-top, and so on) [26] and initial temperature [21]. The Gaussian laser beam whose spot diameter was 80 μm was implemented in SLM280HL.

The track line was formed by the laser irradiation and was cut at the center of the track line using a high precision cut-off machine (RCA-234, Refinetech Co., Ltd., Kanagawa, Japan). Note that the melt pool geometry was evaluated using the one cross-sectional image. Resin was embedded into the cross-sectional surface using a press machine (CitoPress-10, Struers Inc., Cleveland, OH, USA). The specimen was polished using a polishing machine (AutoMet 2000, Buehler Inc., Tokyo, Japan). A colloidal silica solution was added during the final polishing process. After the final polishing, the cross-sectional surface was washed with water, and was sufficiently dried. 

**Figure 3:**
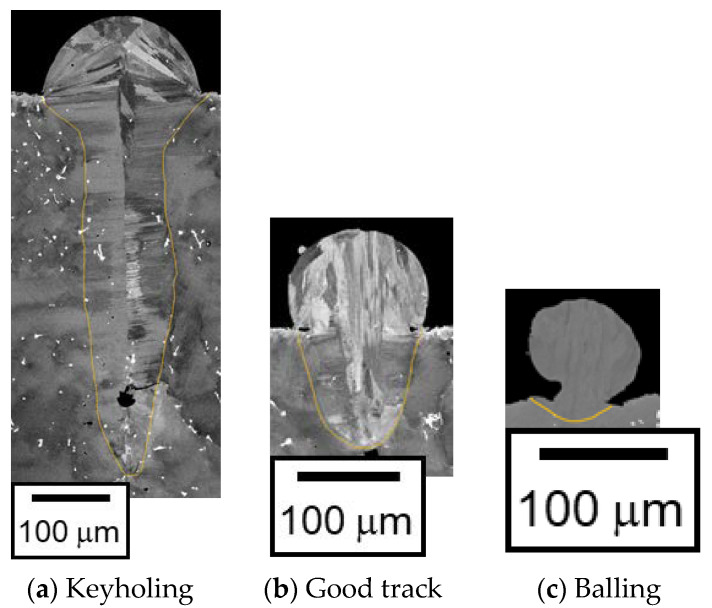
Example images of the single-track experiments: *P* = 300 W, *V* = 250 mm/s (**a**), *P* = 300 W, *V* = 1000 mm/s (**b**), and *P* = 300 W, *V* = 3000 mm/s (**c**).

An example of the cross-sectional image is shown in Figure 1. The melt pool width (*W*) and depth (*D*) are measured from the image. The keyholing, good track, and balling examples are shown in Figure 3a–c, respectively. 

To identify the lack of fusion, the criterion *D* < *t* has been used in past studies [2,11,16]. This means that the melt pool depth does not reach the bottom of the powder layer; thus, porosity must be formed even in the single scan. Following past studies, the lack of fusion criterion of *D* < *t* was used in this study. Note that the criterion *D*_ov_/*t* can be the better criterion for the multi scans. Hence, we discuss a comparison between *D*/*t* and *D*_ov_/*t* in a later section.

According to past studies, keyholing was identified using *D*/*W* > 2.2 [2], *D*/*W* > 1.5 [18], and *D*/*W* > 1.0 [17,18]. Considering the past studies, the keyholing criterion of *D*/*W* > 2.0 was used in this study.

As described in the introduction section, the melt pool length (*L*) is required to identify balling. The melt pool length is hardly measured from the image obtained from the experiment. The track line of the balling forms a distinctive shape; e.g., approximately circular shape in the cross-sectional image, and wave-form from the top views. Hence, balling was identified when such distinctive shapes were observed from the images. The good track was identified when the single-track experiment result did not correspond to keyholing, lack of fusion, or balling.

### 2.2. Finite-Element Analysis

**Figure 4:**
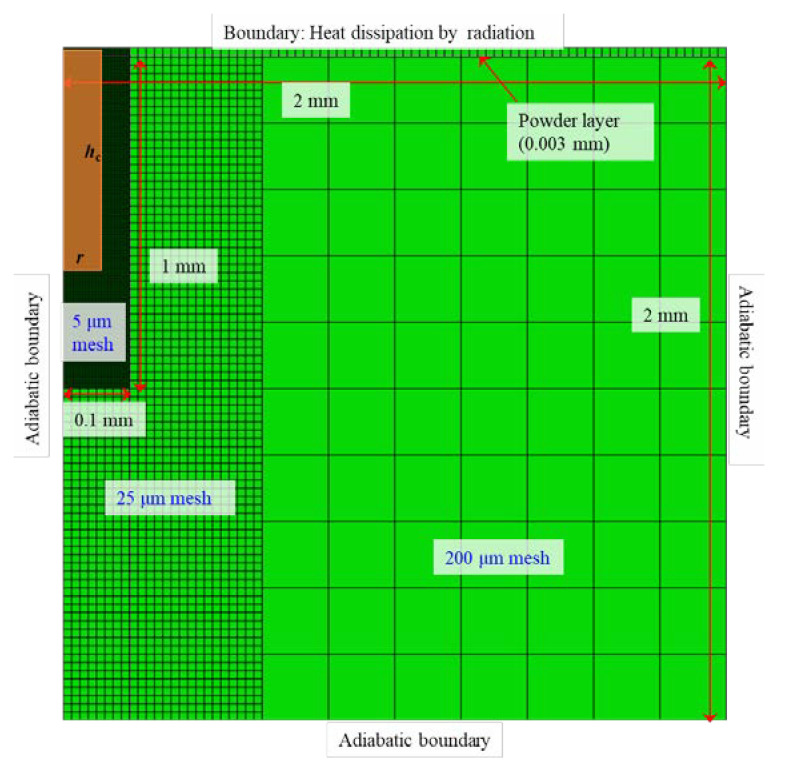
The simulation settings in the two-dimensional thermal conduction FEM analysis.

The two-dimensional thermal conduction analysis with moving heat source model was conducted using a muti-purpose finite element method software: Abaqus 2020. The three-dimensional simulation is of course preferable for simulating the L-PBF process; however, it requires a long computation time. Moreover, many simulations are required to generate the process window; hence, two-dimensional simulation was employed in this study. The computation time of the typical case is approximately 5 min. Figure 4 shows a simulation setting of the FEM analysis. The four-node quadrilateral element was employed in this study. The element was second-order. The total number of elements were 2406. As shown in Figure 4, the mesh size was varied: 5 μm, 25 μm, and 200 μm; 5 μm meshes were placed near a heat source region (brown in Figure 4). Following the default setting in Abaqus software, two convergence criteria were used in this study: (1) the residual of heat flux is less than 0.005 and (2) the temperature correction is less than 0.01. The convergence of FEM simulation using Abaqus is detailed in the software document. 

The powder layer was set at the top 30 μm of the calculation domain. The heat dissipation by radiation condition was adopted to the top surface. The adiabatic boundary condition was applied to the other three boundaries. Under these boundary conditions and 1.0 × 10^−5^ s of the time increment, thermal conduction simulation for 0.005 s was conducted.

**Figure 5:**
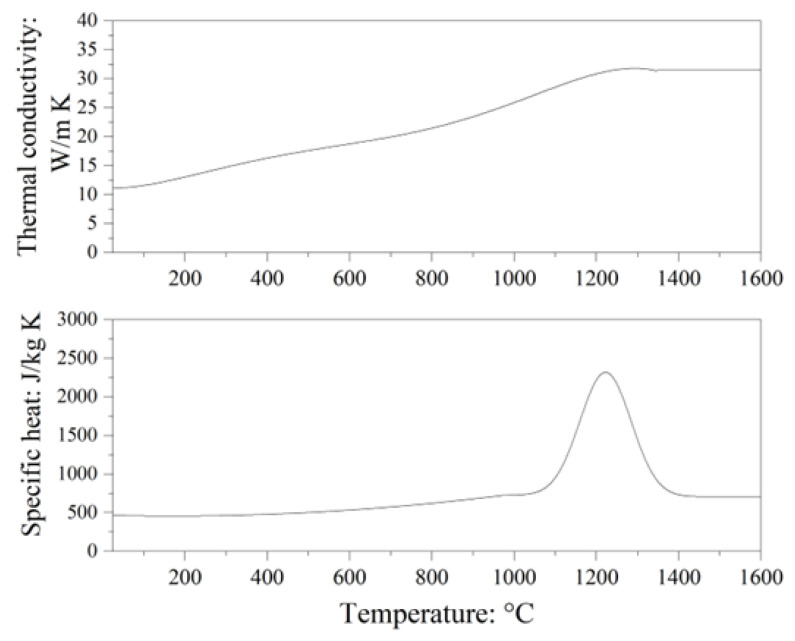
The specific heat and thermal conductivity of Inconel738 LC used in the FEM analysis.

The specific heat and thermal conductivity (*λ*) used are shown in Figure 5. The thermal conductivity of powder layer was *λ*/20. The density of the solid materials and the powder layer were 8220 kg/m^3^ and 4110 kg/m^3^, respectively. The solidus and liquidus temperatures were 1098 °C and 1347 °C, respectively. The latent heat used was 250,000 J/kg. These properties are based on the calculation using the CALPHAD software: JMatPro (https://www.sentesoftware.co.uk/jmatpro (accessed on 7 January 2023)).

The *P* varied between 300 W and 700 W every 100 W, while the *V* varied between 300 mm/s and 4000 mm/s every 100 mm/s; a total of 190 cases was simulated. 

Kusano et al. developed a circular truncated cone shaped heat flux model based on their single-track experiments using Inconel738LC [27]. According to Kusano et al., the simple cylindrical shape can be used instead of the truncated cone. Using the cylinder radius (*r*) and the cylinder height (*h*_c_), the cylindrical model is expressed by the following equations based on [27]:

(1) $$r=38.2-44.5\left(\frac{P}{V}\right),$$

(2) $$h_{c}=-3.3+474.2\left(\frac{P}{V}\right),$$

where *P* and *V* are the laser power and the scan speed, respectively. Due to the two-dimensional analysis in this study, the heat flux was applied to the rectangular region (brown color in Figure 4). The laser heat source moves from the back to the front side of the paper surface in Figure 4. The heat flux (*q*) and the duration time applying the heat flux (*t*_q_) is calculated by the following equations [27]:

(3) $$q=\frac{P\alpha }{4arh},$$

(4) $$t_{q}=\frac{2a}{V},$$

where *a* and *α* are the laser spot radius (40 μm) and the laser absorptivity (0.4 in this study), respectively. Note that the model parameters in Equations (1) and (2) are slightly different from that in [21]. The model in [27] and that in this study are the cylindrical heat source model; however, the *α* in this study is constant, while that in [27] is expressed as the function of *P* and *V*. It should be noted that Equations (1) and (2) can reasonably simulate the melt pool geometry obtained from the single-track experiments though the *α* is constant in this study. 

The calculation procedure for the melt pool depth, width, and length is as follows. When a temperature of a calculation node exceeds the liquidus temperature (*T*_l_), the position of the node (*x*_e_, *y*_e_) is stored. Its maximum values (*x*_e_^max^, *y*_e_^max^) are selected from the nodes whose temperature exceed the liquidus temperature. Using the *x*_e_^max^ and the *y*_e_^max^, the melt pool width (*W*) and depth (*D*) are calculated by the following equations:

(5) $$W=2x_{e}{}^{\max},$$

(6) $$D=y_{e}{}^{\max}.$$

The double track simulation was required to determine *D*_ov_ in Figure 2. In this study, the *D*_ov_ was estimated using two assumptions: (1) the hatching distance (*h*) between the first and second tracks was fixed to 100 μm, and (2) the same melt pools aligned keeping a distance of *h*. Following the study in [2], the *D*_ov_ was calculated by:

(7) $$D_{\mathrm{OV}}=D\left(1-\frac{h^{2}}{W^{2}}\right).$$

The melt pool length (*L*) was estimated using the time when a calculation node first exceeded the liquidus temperature (*t*_min_) and that when a calculation node last exceeded the liquidus temperature (*t*_max_):

(8) $$L=V\left(t_{\max}-t_{\min}\right).$$

The *W*, *D*, *D*_ov_, and *L* were calculated using Equations (5)–(8); as a result, the values of *D*/*W*, *L*/*D*, *D*/*t*, *D*_ov_/*t* were obtained.

Note that a series of the FEM simulations were conducted using a workflow on the Materials Integration System by networking technology (MInt) [28,29,30]. As described above, the FEM simulation requires various simulation parameters including the physical and the laser properties. In the MInt system, the simulation parameters in the input file and the user-defined subroutines for the FEM simulation can be varied through the intuitive GUI interface on the web browser. Moreover, the simulation workflow is designed to input multiple values of the laser properties; as a result of conducting the simulation workflow, the necessary information to generate the process window, i.e., the values of *D*/*W*, *L*/*D*, *D*/*t*, *D*_ov_/*t* under various laser properties, are obtained. A Python application programming interface (API) is implemented in the MInt system; users can conduct the workflow though the Python script. A unique access token is provided to each user, which means that the user can securely access the MInt system. 

## 3. Results and Discussion

### 3.1. Validation of the FEM Analysis

**Figure 6:**
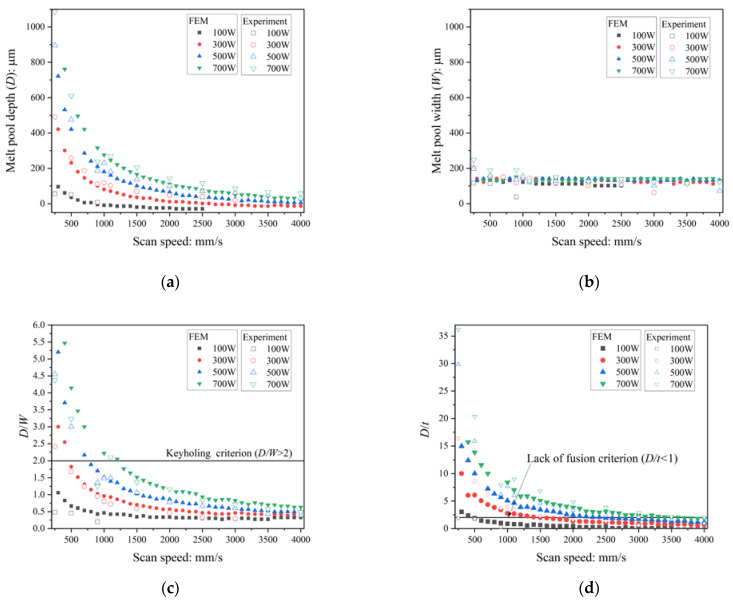
Comparison of melt pool depth: *D* (**a**), width: *W* (**b**), *D*/*W* (**c**), and *D*/*t* (**d**) between the single-track experiments and FEM simulations.

Figure 6 shows the comparison of the melt pool depth and width between the single-track experiment and the FEM analysis. As shown in Figure 6a, the *D* values obtained from the FEM analysis were almost identical to those of the single-track experiments. The *W* values are in good agreement with that of the single-track experiments under *V* > 500 mm/s (Figure 6b). This is because the model parameters in Equations (1) and (2) are based on the single-track experiments for the range of *V* between 750 mm/s and 3500 mm/s. 

As shown in Figure 6c, the *D*/*W* of the experiments and that of the FEM are slightly different mainly because of the difference of *W* for *V* < 500 mm/s. The case of *D*/*W* > 2 arises for *V* < 1000 mm/s. For most cases in *V* < 1000 mm/s, the *D*/*W* of the FEM exceeds 2.0 when that of the experiment also exceeds 2.0, which means that keyholing in the single-track experiments can be identified by the FEM analysis using the criterion of *D*/*W* > 2. The same applies with the discussion in the *D*/*W*, the cases of *D*/*t* < 1 in the FEM when *D*/*t* < 1 in the experiments, as shown in Figure 6d. As the discussions above, the melt pool depth and width in the single-track experiments are approximately modeled by the FEM analysis. 

### 3.2. Balling Criterion

**Figure 7:**
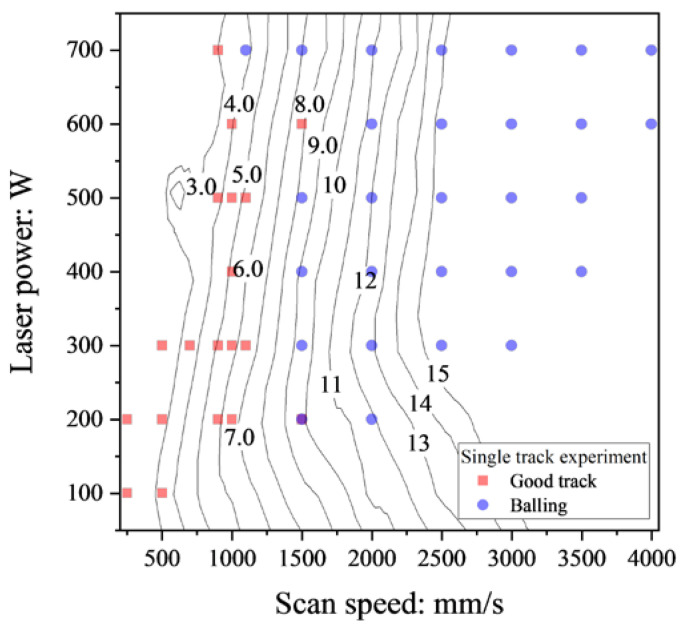
The contour diagram of *L*/*D* obtained by the FEM analysis and the classification of the balling and the good track of the single-track experiments. The boundary between the good track and the balling is between about *L*/*D* = 4 and 9; hence, the *L*/*D* is a good predictor for the balling.

Figure 7 shows the contour diagram for *L*/*D* obtained from the FEM analysis and the classification of the balling and the good track in the single-track experiments.

It should be noted that the balling (blue circle in Figure 7) may plot together with the good track because the balling was identified by the distinctive feature in the cross-sectional image. 

As shown in Figure 7, the boundary between the balling and the good track is in between about *L*/*D* = 4.0 and *L*/*D* = 9.0. The balling arises from the complex, multi-physical process during the L-PBF; hence its influencing factors are not fully clear [3]. Plateau–Rayleigh instability is that the cylindrical fluid stream splits into small droplets and is a potential influencing factor of the balling [2,3,16]. DebRoy et al. suggested that balling can be averted depending on the melt pool length and depth, and proposed the criterion *L*/*D* > π [16]. This criterion is derived from the theoretical analysis in the study of [4]. Let us consider the capillary instability of an infinitely long cylindrical fluid stream whose diameter is *D*_cyl_. The cylinder has axial harmonic disturbances of its diameter with a wavelength of *λ*_cyl_. The cylindrical stream is stable when the *λ*_cyl_ is less than the circumference of the cylinder. The necessary and sufficient condition for Plateau–Rayleigh instability to arise is denoted by π*D*_cyl/_*λ*_cyl_ > 1; this may be the basis of the balling criterion of *L*/*D* > π. 

**Figure 8:**
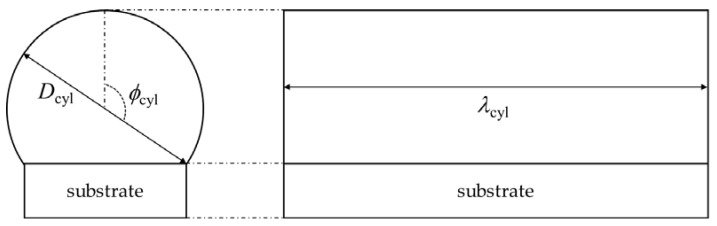
Schematic of a segmented cylindrical melt pool in the study of Gsarov and Smrov [3].

Gusarov and Smurov derived the necessary and sufficient condition for Plateau–Rayleigh instability to arise assuming that the melt pool formed a cylinder partly fixed with the substrate [3]. A schematic of such a segmented cylinder is shown in Figure 8.

Based on Figure 8, the necessary and sufficient condition for Plateau–Rayleigh instability to arise was derived in [3]. The condition is as follows:

(9) $$\frac{\pi D_{\mathrm{cyl}}}{\lambda_{\mathrm{cyl}}}=\sqrt{2}\sqrt{\frac{\phi_{\mathrm{cyl}}\left(1+\cos2\phi_{\mathrm{cyl}}\right)-\sin2\phi_{\mathrm{cyl}}}{2\phi_{\mathrm{cyl}}\left(2+\cos2\phi_{\mathrm{cyl}}\right)-3\sin2\phi_{\mathrm{cyl}}}}.$$

According to Gusarov and Smurov [3], Equation (9) is applicable when *ϕ*_cyl_ > π/2. The calculated values of λ_cyl_/*D*_cyl_ for *ϕ*_cyl_ = π/2, 2π/3, 5π/6 are listed in Table 2.

The *D* value used in the contour map in Figure 7 is the melt pool depth and is different from *D*_cyl_. This means that *λ*_cyl_/*D* is more appropriate than λ_cyl_/*D*_cyl_ for comparing with *L*/*D* in Figure 7. The *D* value in the segmented cylinder in Figure 8 is easily calculated by the geometrical consideration. The relationship between *D* and *D*_cyl_ in the segmented cylinder in Figure 8 is expressed by following equation:

(10) $$D=\frac{1}{2}D_{\mathrm{cyl}}\left(1-\cos\left[\pi -\phi_{\mathrm{cyl}}\right]\right).$$

**Figure 9:**
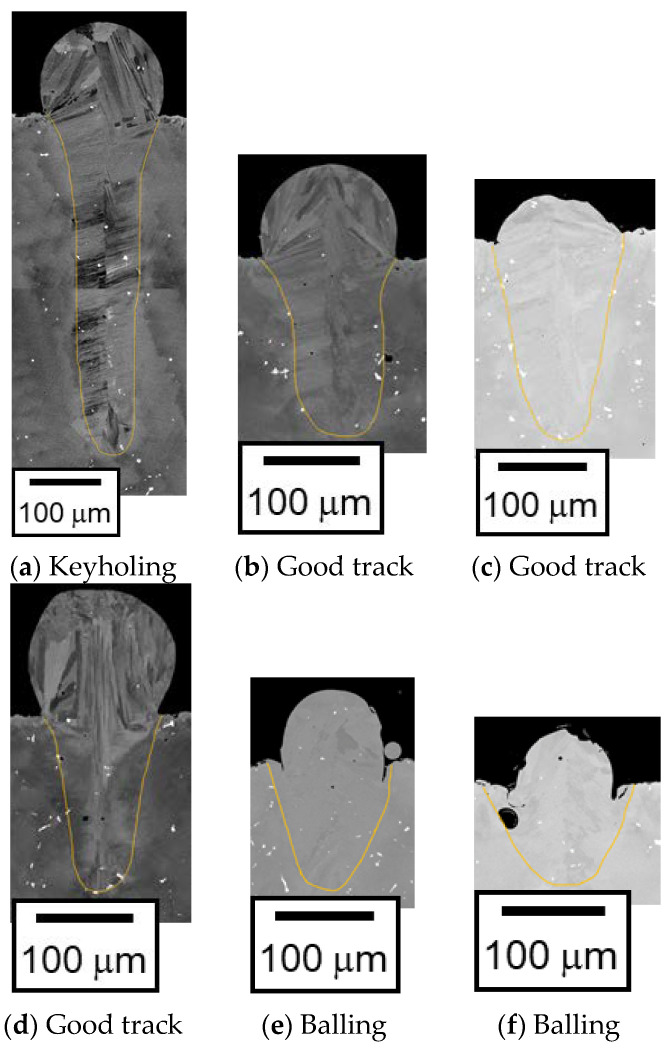
The cross-sectional images in the single-track experiments under *P* = 500 W and *V* = 500 (**a**), 900 (**b**), 1000 (**c**), 1100 (**d**), 1500 (**e**), and 2000 (**f**) mm/s.

The λ_cyl_/*D* values are also listed in Table 2. Assuming that *λ*_cyl_ is equivalent to the melt pool length, *L*, *λ*_cyl_/*D* corresponds to *L*/*D*. Both the good track and the balling are placed in the range between 4 and 9 for *L*/*D* in Figure 7, which implies that the criterion for separating the good track and the balling should be in this range. As shown in Figure 7, the potential criterion is in between 6 and 9 for *L*/*D*. This range is similar to λ_cyl_/*D* = 7.694 when *ϕ*_cyl_ = π/2 rad: the melt pool forms a semi-circle. For the laser power of 500 W, the cross-sectional images of the single-track experiments for *V* = 500 mm/s, 900 mm/s, 1000 mm/s, 1100 mm/s, 1500 mm/s, and 2000 mm/s are shown in Figure 8. The classifications are also displayed in Figure 9. The keyholing defect was observed in *V* = 500 mm/s. The good track and balling defect were found at 900 mm/s to 1100 mm/s and 1500 mm/s to 2000 mm/s, respectively. Note that the other cases were the balling when *V* > 2000 mm/s. Figure 9 implies that the boundary between the good track and the balling should be placed between 1100 mm/s and 1500 mm/s. As shown in Figure 9e,f, the upper part of the melt pool shape (the shape of the melt pool above the substrate) were almost semi-circular shaped, which justifies the use of the segmented cylinder of *ϕ*_cyl_ = π/2 in Table 2. In the typical case of the balling, the melt pool forms a semi-circle or a segmented-circle whose area is larger than that of the semi-circle. Following the discussion above, the criterion *L*/*D* > 7.69 was used for determining the boundary between the good track and the balling.

### 3.3. Lack of Fusion Criterion

**Figure 10:**
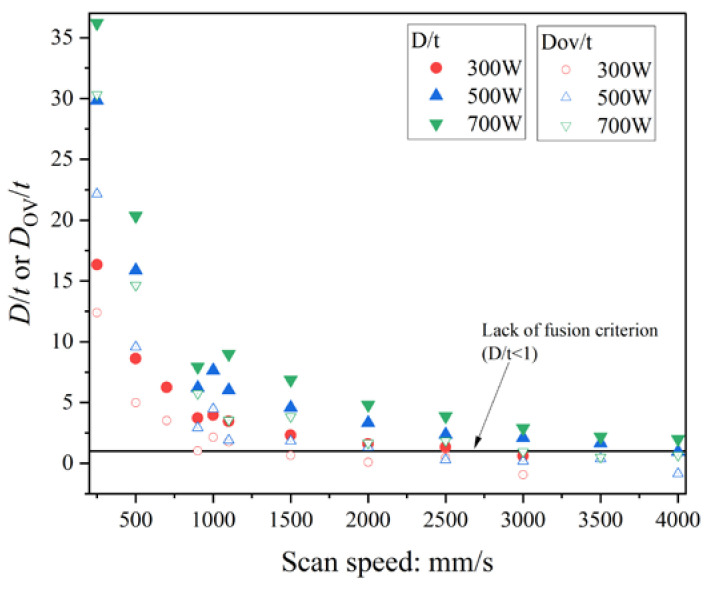
The *D*/*t* and *D*_ov_/*t* estimated assuming that *h* = 100 μm for the single-track experiments.

Figure 10 shows the comparison of *D*/*t* and *D*_ov_/*t* of the single-track experiments. Note that the values of *D*_ov_ were estimated using Equation (7) assuming that *h* = 100 μm. The *D*_ov_ is the depth of the overlap area between the first and the second scans; hence, *D*_ov_ < *D* if *h* > 0. As shown in Figure 10, the *D*_ov_/*t* values are all smaller than *D*/*t*. This means that the *D*_ov_/*t* criterion is required to identify the lack of fusion. Since the single-track experiments were conducted, the true lack of fusion in the muti-scan process could not be observed in this study. In other words, the lack of fusion was identified by the use of *D*/*t* < 1 in the single-track experiments. Therefore, a *D*_ov_/*t* criterion identical to *D*/*t* < 1 was determined in this study.

**Figure 11:**
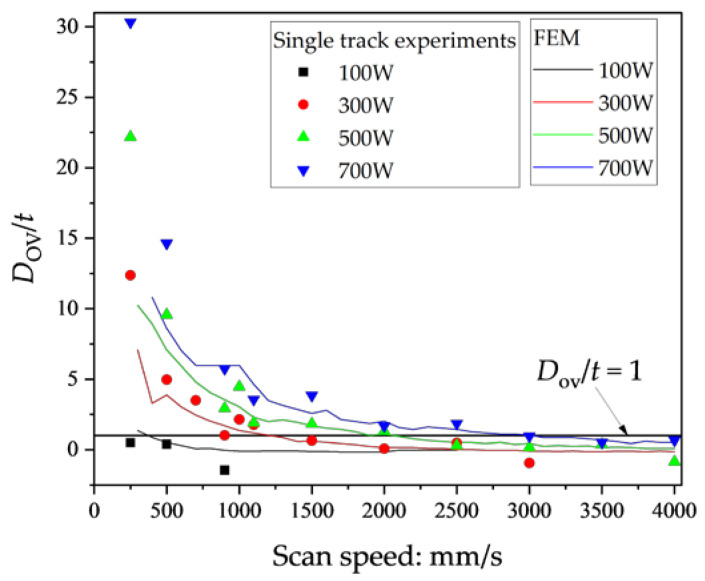
Comparison of *D*_ov_/*t* assuming that *h* = 100 μm obtained from single-track experiments and the FEM analysis.

Figure 11 shows the comparison of *D*_ov_/*t* assuming that *h* = 100 μm obtained from the single-track experiments and the FEM analysis. When *V* > 1000 mm/s, *D*_ov_/*t* obtained from the single-track experiments were almost identical to those from the FEM analysis, while the difference of the single-track experiments and the FEM analysis increased with the decrease of *V* when *V* < 1000 mm/s. When using the criterion *D*/*t* < 1, the *D*_ov_/t criterion should be smaller than 1 because *D*_ov_ < *D*. As shown in Figure 11, the *D*_ov_/*t* obtained from the single-track experiments hardly differs from that from the FEM analysis when *D*_ov_/*t* < 1; hence, the *D*_ov_/*t* criterion was estimated from the contour map generated from the FEM analysis.

**Figure 12:**
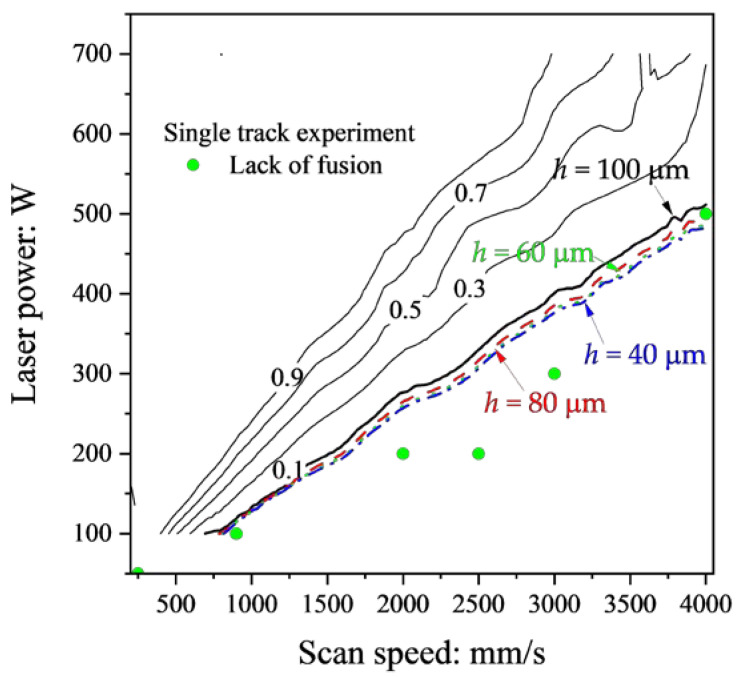
The contour lines of *D*_ov_/*t* using *h* = 100 μm (black) and the isolines of *D*_ov_/*t* = 0.1 using *h* = 100 μm (black, bold), 80 μm (red), 60 μm (green), and 40 μm (blue) obtained by the FEM analysis and the classification of the lack of fusion of the single-track experiments.

Figure 12 shows the contour map of *D*_ov_/*t* using *h* = 100 μm (black lines), and the isolines of *D*_ov_/*t* = 0.1 using *h* = 100 μm (black), 80 μm (red), 60 μm (green), and 40 μm (blue) obtained from the FEM analysis. The lack of fusion in Figure 12 was classified using *D*/*t* < 1. As can be seen in the contour map in Figure 12, the boundary between the balling and the lack of fusion is classified by *D*_ov_/*t* = 0.1 using *h* = 100 μm. Although the isolines of *D*_ov_/*t* = 0.1 cross a minimal region of the balling, the criterion *D*_ov_/*t* = 0.1 can reasonably identify the lack of fusion. Moreover, the isolines of *D*_ov_/*t* = 0.1 hardly differ irrespective of the *h*. The plots of the lack of fusion in Figure 12 were identified using *D*/*t* < 1, i.e., *D*_ov_/*t* < 0.1, the criterion identical to *D*/*t* < 1.

**Figure 13:**
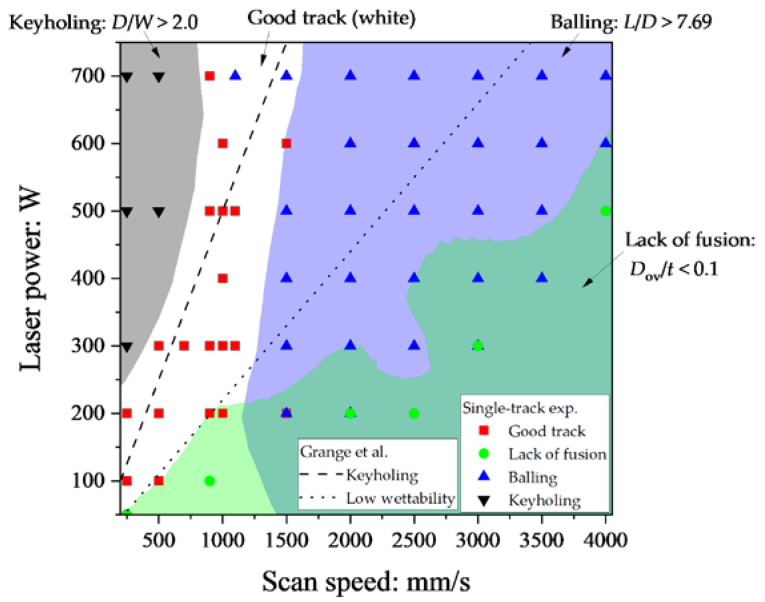
The process window of Inconel738LC using the criteria *D*/*W* > 2.0, *L*/*D* > 7.69, and *D*_ov_/*t* < 0.1.

The process window using the criteria of *D*/*W* > 2.0, *L*/*D* > 7.69, and *D*_ov_/*t* < 0.1 (*D*/*t* < 1) is shown in Figure 13. The regions of *D*/*W* > 2 (gray) and *D*_ov_/*t* < 0.1 (green) were generated from the contour maps of the results of the single-track experiments. Moreover, the *D*_ov_ was calculated assuming that *h* = 100 μm. The region of the lack of fusion in Figure 13 was different from that of *D*_ov_/*t* < 0.1 in Figure 12 because the isoline of *D*_ov_/*t* = 0.1 in Figure 12 was based on the results of the FEM simulations. The region of *L*/*D* > 7.69 (blue) was based on the results of the FEM simulations. The keyholing and the balling were well classified using *D*/*W* > 2.0 and *L*/*D* > 7.69, respectively. On the other hand, *D*_ov_/*t* < 0.1 strictly agreed with the experimental classification. The reason for the disagreement should be the small amount of the lack of fusion data. The number of instances of lack of fusion was just 6 out of a total of 68 experiments. The criterion of *D*_ov_/*t* should be verified when the number of lack of fusion data is increased. Grange et al. conducted single-track experiments using Inconel738LC and classified the keyholing and a low wettability using the boundaries of *P*/*V* = 0.5 J/mm and *P*/*V* = 0.22 J/mm, respectively [23]. The keyholing area in this study was smaller than that in [23] because of the difference of criterion between this study: *D*/*W* > 2 and [23]: *D*/*H*_app_ > 3. *H*_app_ is the height of the upper part of the melt pool and is similar to the powder layer thickness: *t* in Figure 1. The low wetting boundary crossed the balling region in Figure 13. The low wettability is the area of *P*/*V* < 0.22 J/mm and its feature is small *D* and high wetting angle (*θ* > 90°). As shown in Figure 1, the wetting angle tends to be high in the balling in this study, which means that the low wettability has features of the lack of fusion and the balling. The proposed method can quantitatively classify the lack of fusion and the balling: this is an advantage of this study.

Here, the whole procedure to generate the process window of Inconel738LC was demonstrated. The simulation workflow is easily applied to other alloys; this is an advantage of the simulation workflow on the Mint system. It should be noted that the criteria of *D*/*W*, *L*/*D*, and *D*_ov_/*t* used in this study may not be appropriate for the other materials; hence, such criteria are preliminary determined before applying the simulation workflow.

## 4. Conclusions

In this study, the criteria for identifying the balling and the lack of fusion of Inconel738LC were investigated using single-track experiments and FEM analysis of thermal conduction. The following results were obtained.

- Using the melt pool depth (*D*), width (*W*), the powder layer thickness (*t*), and the overlap depth between the first and the second scans (*D*_ov_) assuming the hatching distance (*h*) of 100 μm, the experimental results were classified into three defect types: keyholing, lack of fusion, and balling by *D*/*W* > 2.0, *D*/*t* > 1, and the distinctive feature of the cross-sectional image and top view, respectively.
- The FEM analysis was validated by comparing the *D* and *W* between the single-track experiments and the FEM analysis. As a result, the melt pool geometry obtained from FEM analysis reasonably agreed with that from the single-track experiments.
- A series of FEM analyses was conducted varying the laser power and the scan speed. The contour map of the ratio of melt pool length and depth (*L*/*D*) was generated from the FEM analysis. Considering the necessary and sufficient condition arising Plateau–Rayleigh capillary instability which is the main cause of the balling, the balling criterion of *L*/*D* > 7.69 was obtained from the comparison with the contour map and the classification of the single-track experiments.
- The *D*_ov_/*t* assuming *h* = 100 μm was calculated. As a result, the lack of fusion criterion of *D*_ov_/*t* < 0.1 which was equivalent to the well-known criterion: *D*/*t* < 1 was obtained.
- Finally, the process window of Inconel738LC was generated using the criteria of *D*/*W* > 2.0, *L*/*D* > 7.69, and *D*_ov_/*t* < 0.1.

An important outcome of the procedure to generate the process window is that the balling defect can be quantitatively determined by *L*/*D* > 7.69. As a result, keyholing, lack of fusion, and balling can be identified by a series of FEM simulations if the criteria determined can be applied to a target material. However, the material used is Inconel738LC only, which means that the criteria determined are valid for Inconel738LC. According to past studies [17], the thermal properties of Hastelloy X are similar to those of Inconel738LC. Assuming that the melt pool geometry mainly depends on the thermal properties, there is a possibility of using the criteria determined in this study for the other materials. The validity of the criteria determined against the other materials will be investigated in our future study. Moreover, the criterion *D*_ov_/*t* < 0.1 is identical to *D*/*t* < 1, which seems to be the minimum requirement of the lack of fusion for the single scan. In order to evaluate the lack of fusion in the multi scans, the *D*_ov_/*t* values will be evaluated by multi-track experiments and CFD simulations in our future work. 

## Figures and Tables

**Table 1:** The laser power and the scan speed (the translation velocity of the moving laser) in the single-track experiments.

Laser Power: W	Scan Speed: mm/s
50	250, 500, 900, 1100, 1500, 2500, 3500
100	250, 500, 900, 1100, 1500, 2500, 3500
200	250, 500, 900, 1000, 1100, 1500, 2000, 2500, 3500
300	250, 500, 700, 900, 1000, 1100, 1500, 2000, 2500, 3000, 3500
400	1000, 1500, 2000, 2500, 3000, 3500
500	250, 500, 900, 1000, 1100, 1500, 2000, 2500, 3000 3500 4000
600	1000, 1500, 2000, 2500, 3000, 3500, 4000
700	250, 500, 900, 1100, 1500, 2000, 2500, 3000, 3500, 4000

**Table 2:** The *ϕ*_cyl_, λ_cyl_/*D*_cyl_, melt pool depth, and *λ*_cyl_/*D* of a segmented cylindrical melt pool model shown in Figure 8.

*ϕ*_cyl_: deg. (Rad)	*λ*_cyl_/*D*_cyl_	Melt Pool Depth *D*: μm	*λ*_cyl_/*D*
90 (π/2)	3.847	0.5*D*_cyl_	7.694
120 (π/3)	4.786	0.25*D*_cyl_	19.14
150 (π/6)	4.113	0.067*D*_cyl_	61.64
180 (π)	3.847	0	No overlap

## Data Availability

Data in this manuscript are not available.
